# Influence of beam hardening in dual-energy CT imaging: phantom study for iodine mapping, virtual monoenergetic imaging, and virtual non-contrast imaging

**DOI:** 10.1186/s41747-021-00217-1

**Published:** 2021-04-27

**Authors:** Risa Kanatani, Takashi Shirasaka, Tsukasa Kojima, Toyoyuki Kato, Masateru Kawakubo

**Affiliations:** 1 Department of Health Sciences, School of Medical Sciences, Kyushu University, Fukuoka, Japan; 2 Department of Radiology, Saiseikai Fukuoka General Hospital, Fukuoka, Japan; 3 Division of Radiology, Department of Medical Technology, Kyushu University Hospital, Fukuoka, Japan; 4 Department of Health Sciences, Graduate School of Medical Sciences, Kyushu University, Fukuoka, Japan; 5 Department of Health Sciences, Faculty of Medical Sciences, Kyushu University, Fukuoka, Japan

**Keywords:** Iodine, Phantoms (imaging), Tomography (x-ray, computed), x-rays

## Abstract

In this study, we investigated the influence of beam hardening on the dual-energy computed tomography (DECT) values of iodine maps, virtual monoenergetic (VME) images, and virtual non-contrast (VNC) images. 320-row DECT imaging was performed by changing the x-ray tube energy for the first and second rotations. DECT values of 5 mg/mL iodine of the multi-energy CT phantom were compared with and without a 2-mm-thick attenuation rubber layer (~700 HU) wound around the phantom. It was found that the CT density values UH, with/without the rubber layer had statistical differences in the iodine map (184 ± 0.7 *versus* 186 ± 1.8), VME images (125 ± 0.3 *versus* 110 ± 0.4), and VNC images (−58 ± 0.7 *versus* −76 ± 1.7) (*p* < 0.010 for all). This suggests that iodine mapping may be underestimated by DECT and overestimated by VME imaging because of x-ray beam hardening. The use of VNC images instead of plain CT images requires further investigation because of underestimation.

## Key points

- The influence of x-ray beam hardening on dual-energy computed tomography (CT) was investigated.
- CT values of iodine mapping may be underestimated because of x-ray beam hardening.
- CT values of virtual monoenergetic imaging may be overestimated because of x-ray beam hardening.
- Further investigation may be required for the use of virtual non-contrast images as an alternative to plain CT.

## Background

Dual-energy computed tomography (DECT) is an imaging technique that utilizes continuous x-ray energies at two different kVp to generate iodine maps, virtual monoenergetic (VME) images, and virtual non-contrast (VNC) images. An iodine map can be used to identify the contrast media of iodine in any anatomical region [1–4]. Moreover, VME imaging, which refers to the generation of virtual images scanned at lower or higher actual effective tube voltages relative to the conventional scan voltage, can be applied to improve the enhancement of contrast media or reduce metal artifacts [5, 6]. Further, the VNC method reduces the need for plain computed tomography (CT) scans and also reduces patient radiation exposure, as VNC images are virtually reconstructed from the DECT images [7]. The calculations to obtain these novel CT images are based on the differences in the attenuation coefficient values of materials at the low and high effective x-ray energies utilized for DECT scanning [8]. However, in clinical examinations, x-rays can cause a beam-hardening effect due to differences in the amount of x-ray transmitted through the human tissue; therefore, the effective incident x-ray energy on the x-ray detector differs from the output x-ray energy [9, 10]. Because lower-energy photons are absorbed more rapidly than higher-energy photons, the spectrum of the x-ray beam becomes more intense by the time the x-ray reaches the detector. This effect may reduce the accuracy of CT values in both low- and high-energy CT images. Thus, it may also affect the accuracy of iodine maps, VME images, and VNC images.

Whereas beam-hardening correction has been implemented in the calculation of DECT imaging [11], to the best of our knowledge, no published study has investigated the influence of x-ray beam hardening on the CT values of various materials. Consequently, in this study, we investigated the effect of beam hardening on the DECT values of the iodine map, VNC images, and VME images.

## Methods

### Experiment

**Fig. 1:**
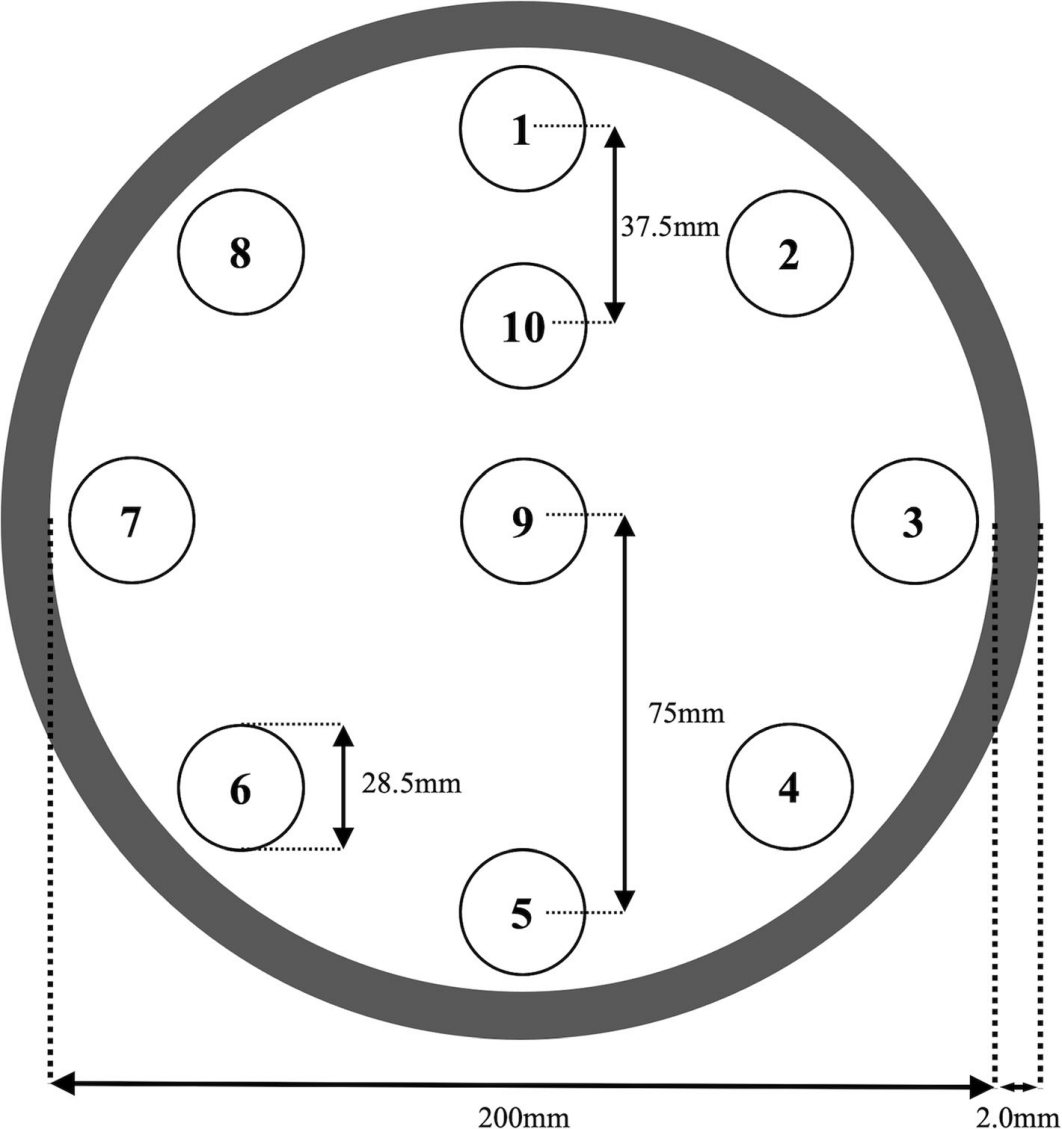
Schematic of multi-energy computed tomography (CT) phantom. The CT scans were performed with and without the x-ray attenuation rubber layer (width 2.0 mm, indicated as a gray layer)

This study was a phantom experiment and did not require ethics committee approval. We used a 320-row CT scanner (Aquilion One Vision Edition, Canon Medical Systems, Japan) to scan a phantom (Model 1472, Gammex RMI, Middleton, WI, USA) to obtain DECT images. The CT equipment used in this study was calibrated with specific phantoms daily for the quality control. The DECT images were obtained as two datasets, corresponding to the two x-ray tube energies used for the first and second rotations. As shown in Fig. 1, the phantom consisted of a cylinder with a diameter of 200 mm. A total of ten 28.5-mm diameter holes were created, with the resulting background CT value being equivalent to that of water; the holes were structured such that they could be filled with rods of various substances. By assuming brain CT imaging, 10 targets were set; Target_1, Target_3, and Target_7: iodine concentration [mg/mL] of 10.00, 2.00, and 5.00; Target_2, Target_5, Target_6, Target_9, and Target_10: soft tissues as blood (*ρ*_e_^w^ = 1.07), brain (*ρ*_e_^w^ = 1.02), blood (*ρ*_e_^w^ = 1.10), water (*ρ*_e_^w^ = 1.00), and water (*ρ*_e_^w^ = 1.00); Target_4 and Target_8: mixture of iodine and blood (2.00 mg/mL + blood and 4.00 mg/mL + blood). Furthermore, a 2-mm-thick rubber material with a radiodensity of ~700 HU was wound around the cylindrical phantom to function as a high x-ray attenuation layer.

### Scan parameters

The center of the phantom was aligned to coincide with the center of the gantry. The DECT scan parameters were as follows: collimation, 320 × 0.5 mm; gantry rotation time, 1.0 s; tube potential/tube current, 80 kVp/800 mA and 135 kVp/520 mA; *z*-coverage, 160 mm; scan field of view, 24.0 cm. High tube current was used to suppress the fluctuation of the CT value, and the scan was performed nine times for every 36° to change the start position of the x-ray tube. The images were reconstructed by means of adaptive iterative dose reduction (AIDR 3D STD) with the following reconstruction parameters: slice thickness, 2.0 mm; reconstruction kernel, FC43; display field of view, 24.0 cm. The scan field of view and display field of view were set by assuming a head CT examination. To investigate the effects of x-ray beam hardening, we repeated this DECT scan protocol identically with and without the x-ray attenuation rubber layer.

### Image reconstruction

DECT postprocessing was performed by raw data-based decomposition using a dedicated workstation with the CT scanner. The calculation algorithm for the iodine map was based on the three-material decomposition technique using the difference in the attenuation coefficient specific to a substance upon scanning at two tube voltages [12]. For the VME images, a 70-keV monoenergetic beam was simulated to obtain a “conventional CT” impression (comparable to 120-kV images) [13]. The VNC images were obtained by subtracting the iodine maps from the VME images.

### Image analysis

**Fig. 2:**
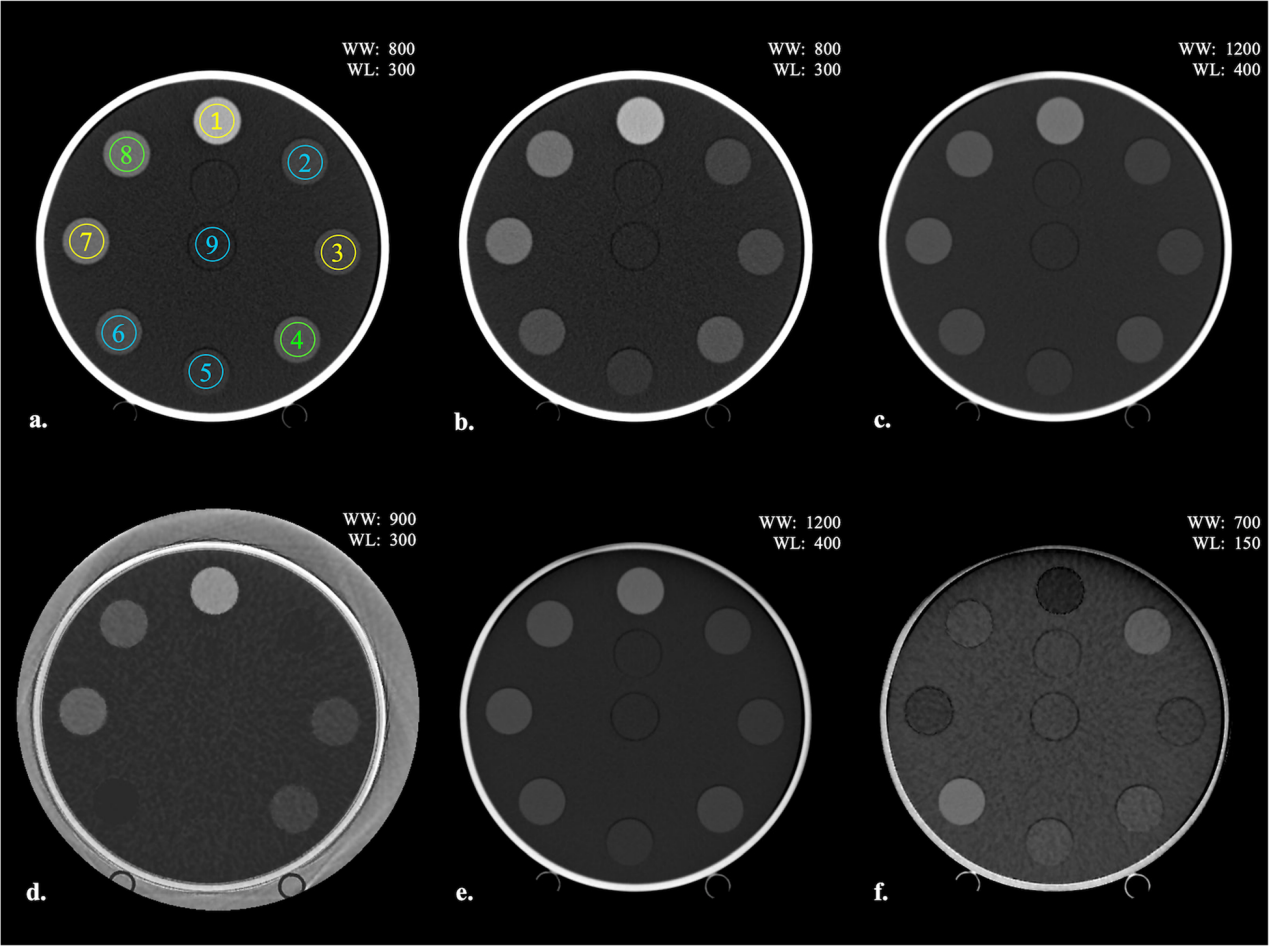
Positions of the regions of interest (ROIs) for measuring computed tomography (CT) values in multi-energy CT phantom image and CT images. **a** All ROIs lie in the same area, which corresponds to 70% of the regional area. Assuming brain CT imaging, 9 ROIs were set; ROI_1, ROI_3, and ROI_7: Iodine concentration [mg/mL] of 10.00, 2.00, and 5.00 (yellow circles); ROI_2, ROI_5, ROI_6, and ROI_9: Soft tissues as the blood (*ρ*_e_^w^ = 1.07), brain (*ρ*_e_^w^ = 1.02), blood (*ρ*_e_^w^ = 1.10), and water (*ρ*_e_^w^ = 1.00) (blue circles); ROI_4 and ROI_8: Mixture of the iodine and blood (2.00 mg/mL + the blood and 4.00 mg/mL + blood) (green circles). **b** Single-energy image (80 kVp). **c** Single-energy image (135 kVp). **d** Iodine map. **e** Virtual monoenergetic image, *F* Virtual non-contrast image

The CT values of the iodine map, VME, VNC, and conventional single low/high energy (80/135 kVp) images were measured as the average values of the circular region of interest (ROI) using ImageJ (free software developed by National Institutes of Health, USA); the ROI comprised 70% of the area of each material. As shown in Fig. 2a, for brain CT imaging, the nine ROIs were randomly set as follows: pure water (1.00 mg/mL); brain (*ρ*_e_^w^ = 1.02); blood (*ρ*_e_^w^ = 1.07 and 1.10); iodine (mg/mL) concentrations of 2.00, 5.00, and 10.00; and mixtures of blood and iodine (mg/mL) concentrations of 2.00 and 4.00. The CT images are shown in Fig. 2b–f.

### Statistical analysis

The normality of the distribution of the measured CT value was determined by the Shapiro-Wilk test, and the descriptive statistical values, specifically the means and standard deviations, were calculated. Because all values were distributed normally, mean CT values of each region over the nine repeated scans were compared by means of a paired *t* test between with and without the x-ray attenuation rubber layer. All statistical analyses were conducted using GraphPad Prism (version 8.1.2 for Mac OS, GraphPad Software, La Jolla, California, USA) with the statistical significance set at *p* < 0.05.

## Results

Table 1 shows a comparison of the results of the CT values with and without the x-ray attenuation rubber layer for the iodine map, VME images equivalent to 70 keV, and VNC images, and the 80/135 kVp single-energy CT images. The CT values of the iodine map and the 80 kVp single-energy CT images without the x-ray attenuation rubber layer were mostly larger than those with it. However, there were no statistical differences in the iodine map for a comparison between iodine (2.00 mg/mL) and blood (1.10 *ρ*_e_^W^). Moreover, the relationships were reversed in the regions of the mixture (2.00 mg/mL iodine + blood), brain, and water in the iodine map and in the regions of the blood, brain, and water in the 80 kVp single-energy CT images. In contrast, in the comparison of VME and VNC images and 135 kVp single-energy CT images, the CT values without the rubber layer were smaller than those with the rubber layer. There were no statistical differences in the regions of the mixture (2.00 mg/mL iodine + blood), brain, and water.

**Table 1:** Comparison of the CT values with and without the x-ray attenuation rubber layer

	Iodine map		VME (70 keV)		VNC		80 kVp		135 kVp	
	Without rubber	With rubber	Without rubber	With rubber	Without rubber	With rubber	Without rubber	With rubber	Without rubber	With rubber
**Iodine (2.00 mg/mL)**	83.7 ± 1.6	84.4 ± 1.0	**31.4 ± 0.4**	**42.3 ± 0.4**	**-52.3 ± 1.6**	**-42.1 ± 0.8**	**101.5 ± 0.3**	**99.9 ± 0.4**	**38.8 ± 0.2**	**46.3 ± 0.3**
**Iodine (5.00 mg/mL)**	**185.7 ± 1.8**	**183.5 ± 0.7**	**110.2 ± 0.4**	**125.2 ± 0.3**	**-75.5 ± 1.7**	**-58.3 ± 0.7**	**233.7 ± 0.4**	**221.4 ± 0.5**	**113.8 ± 0.1**	**118.6 ± 0.2**
**Iodine (10.00 mg/mL)**	**369.1 ± 2.1**	**354.6 ± 0.9**	**218.0 ± 0.5**	**248.5 ± 0.4**	**-151.1 ± 2.0**	**-106.1 ± 0.9**	**460.1 ± 0.4**	**431.1 ± 0.4**	**214.2 ± 0.5**	**222.3 ± 0.5**
**Iodine (2.00 mg/mL) + Blood**	**77.1 ± 1.1**	**80.8 ± 0.6**	**82.8 ± 0.4**	**87.5 ± 0.2**	5.8 ± 1.2	6.8 ± 0.7	**145.3 ± 0.2**	**143.1 ± 0.3**	**89.9 ± 0.3**	**91.1 ± 0.3**
**Iodine (4.00 mg/mL) + Blood**	**159.6 ± 2.5**	**151.7 ± 1.1**	**108.3 ± 0.5**	**135.2 ± 0.3**	**-51.3 ± 2.2**	**-16.5 ± 0.9**	**241.1 ± 0.4**	**229.4 ± 0.5**	**114.6 ± 0.4**	**132.0 ± 0.2**
**Blood (1.07** ***ρ***_**e**_^**W**^**)**	**9.7 ± 1.5**	**6.7 ± 0.7**	**31.3 ± 0.7**	**52.7 ± 0.4**	**21.6 ± 1.0**	**46.0 ± 0.7**	**87.3 ± 0.3**	**90.6 ± 0.3**	**41.6 ± 0.3**	**63.3 ± 0.4**
**Blood (1.10** ***ρ***_**e**_^**W**^**)**	0.6 ± 0.2	0.5 ± 0.2	**92.2 ± 0.6**	**99.0 ± 0.3**	**91.6 ± 0.6**	**98.6 ± 0.4**	**115.8 ± 0.6**	**119.3 ± 0.3**	**102.7 ± 0.3**	**110.3 ± 0.3**
**Brain (1.02** ***ρ***_**e**_^**W**^**)**	**15.0 ± 1.2**	**18.4 ± 1.1**	**22.1 ± 0.3**	**25.2 ± 0.2**	7.1 ± 1.3	6.8 ± 1.0	**43.5 ± 0.5**	**47.5 ± 0.4**	**30.4 ± 0.2**	**34.8 ± 0.3**
**Water (1.00** ***ρ***_**e**_^**W**^**)**	**12.5 ± 0.8**	**15.1 ± 0.7**	**-16.0 ± 0.2**	**-13.2 ± 0.3**	**-**28.5 ± 0.8	-28.3 ± 0.8	**-1.8 ± 0.4**	**0.7 ± 0.4**	**-8.1 ± 0.2**	**-4.3 ± 0.2**

Unless otherwise indicated, data are the mean ± standard deviation. *VME* Virtual monoenergetic, *VNC* Virtual non-contrast. Statistical differences (*p* < 0.05) are underlined for comparison

## Discussion

The CT values of the iodine map with the x-ray attenuation rubber layer were smaller than the corresponding values for the case without the rubber layer. This result indicates that underestimation of the iodine CT values occurred because of x-ray beam hardening. In some regions, there was no difference, or the magnitude relationship was reversed in the iodine map. However, there is a difference between the reference CT values of the water region in the low- and high-single-energy CT images, and the difference values are less than 10 HU in all regions. Therefore, these differences in CT values can be considered as a category of the fundamental error of CT imaging. In the VME images, the CT values with the x-ray attenuation rubber were larger than the corresponding values without the rubber layer. At first glance, this result appears to negate the influence of beam hardening. The VME image in this experiment was virtually reconstructed by using 70 keV x-ray energy assuming that conventional CT imaging uses 120 kVp. Focusing on the CT values of the VME images, the CT values obtained with the rubber layer are within the range of the CT values of low- and high-single CT images, but the CT values without the rubber layer do not show this tendency. Moreover, the CT values of the VNC images with the rubber layer were larger than the corresponding values without the rubber layer. The VNC CT values were obtained by subtracting the iodine-map CT values from the corresponding values of the VME images. Therefore, these results may be attributed to the fact that the contrast improvement in the VME images with the x-ray attenuation rubber exceeded the underestimation of the CT values due to beam hardening in the iodine map.

**Fig. 3:**
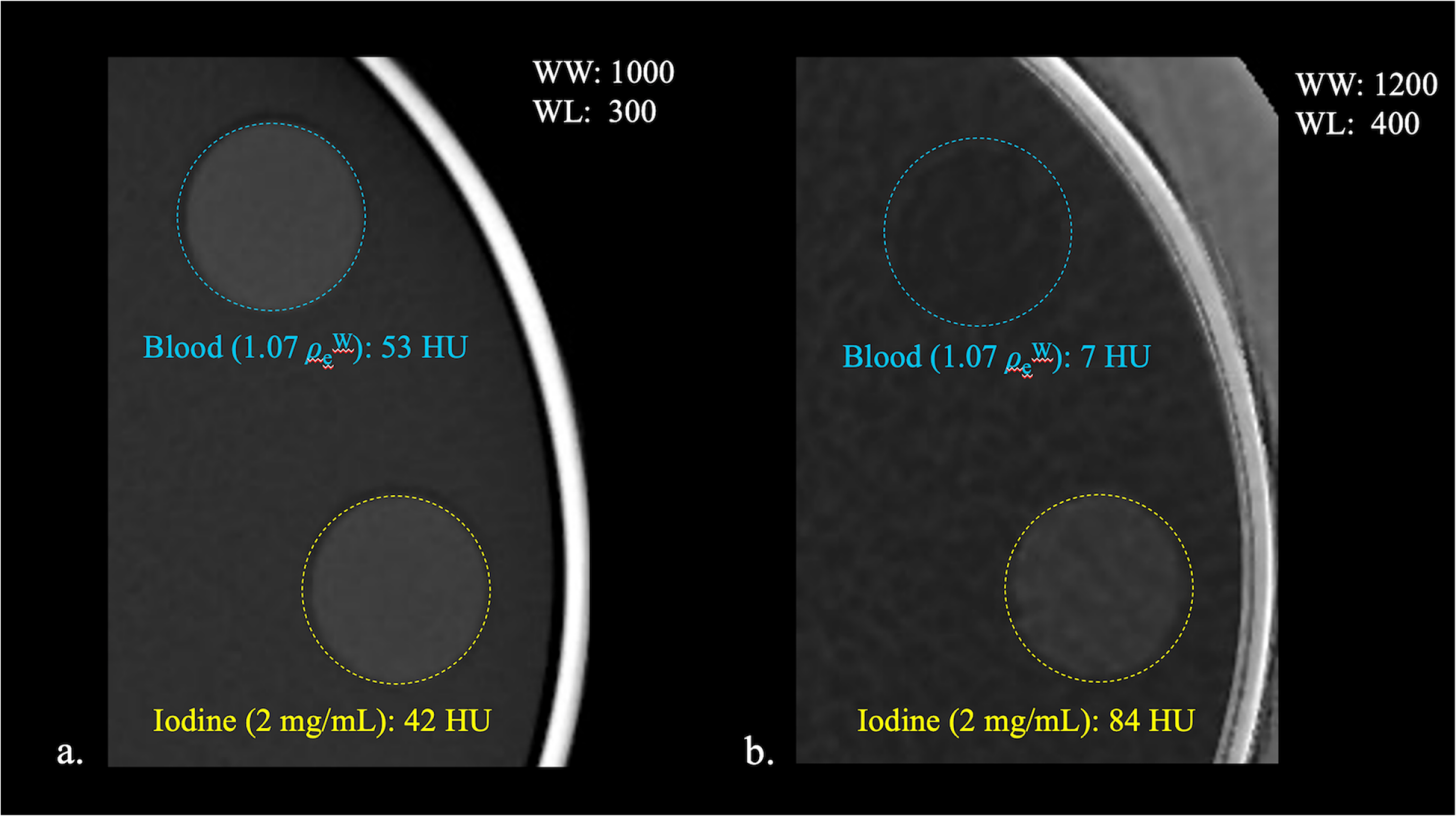
Representative phantom images with x-ray attenuation rubber covering with a focus on the difference between blood (blue dashed circle) and iodine (2.00 mg/mL) (yellow dashed circle). **a** There is almost no visual difference between the blood and iodine in the virtual monoenergetic (VME) image. **b** The two regions can be clearly differentiated in the iodine map

In our present study, when we focused on the differentiation of the blood and 2.00 mg/mL of iodine, which corresponds to equivalent CT values of the blood and iodine in conventional 120-kV CT imaging, the differences in the CT values of the iodine map were > 70 HU regardless of the presence of the rubber layer. In contrast, the underestimation of the CT values of the iodine map, which appears to be due to beam hardening, was as small as 15 HU at the most. Consequently, as regards the clinical use of iodine maps for brain imaging, the underestimation of the CT values of the iodine map is too small to affect the diagnosis. Representative phantom images with the x-ray attenuation rubber layer, with the focus on the visual difference between blood and iodine (2.00 mg/mL) are shown in Fig. 3. There is almost no difference visually between blood and iodine in the VME image; however, these two components can be clearly differentiated in the iodine map.

Upon considering the CT values of water (0 HU) in each image, we note that the iodine map and VME and VNC images show no regions corresponding to CT values of 0 HU. Currently, VNC images are expected to provide information equivalent to plain CT without the need for an actual CT scan, which also implies reduced radiation exposure of the patient [7]. In particular, VNC imaging is expected to greatly reduce the exposure dose of patients who need to repeatedly undergo dynamic CT in a relatively short period of time [14]. However, as our results show, the CT values of water in VNC images may exhibit acceptable error levels because the VNC image is calculated by including both the errors of the iodine map and the VME image. In addition, there are many reports that CT values of VNC, not limited to those of water, are different from true non-contrast CT values [15, 16]. Furthermore, there are reports that VNC underestimates the CT values of calcification and metal concentration [17, 18]. Therefore, we consider that the clinical application of VNC imaging over plain CT imaging may require further investigations.

Our phantom study has certain limitations. Firstly, when applying the rubber layer, we assumed only a simplistic x-ray attenuation with only the phantom consisting of a 200-mm diameter. As regards clinical CT imaging, the human anatomical structure is more complex, and x-ray beam hardening is affected by the size of the objects for CT scan. Because most CT systems with multiple detectors generally acquire whole-brain images with one or two rotations, the beam exhibits a spread along the body axis, which leads to beam hardening in the scan area. Therefore, we could not reproduce clinically exact beam hardening in the study. However, our results reveal that beam hardening affects at least the CT values of DECT. In addition, the differences in CT values depend on the DECT data collection method, which is not considered here. For example, although we selected the scan field of view as 24.0 cm (assuming a head CT examination) in this research, the effect of beam hardening correction depends on the beam filtration setting (*e.g.,* bowtie filter), which is determined by the size of the scan field of view. Further, the effective tube voltage of the CT system used in this research is lower than those provided by other vendors’ CT systems [19, 20]. Therefore, although the CT system used in this research may have stronger beam hardening than that in other vendors’ systems, this limitation is not considered here. Moreover, the DECT imaging approach used in this study was a simple method in which the x-ray tube rotates twice for each of the two energy levels. This method may lead to problems such as misregistration due to the motion of the patient and increased exposure doses in clinical practice. However, we used a simple method involving two repeated scans with the two energy levels; thus, the influence of beam hardening could be clearly examined. Further, the CT values of VME images were not compared to conventional CT images obtained at 120 kVp x-ray energy. Future studies will need to focus on accumulating and reviewing clinical cases and examining the effects of beam hardening under clinical conditions.

In conclusion, in this study, we investigated the influence of x-ray beam hardening on DECT imaging by using an experimental phantom. Statistical differences were found in the CT values between each scan location with and without the rubber layer for the iodine map, VME images, and VNC images. These results imply that the iodine map acquired with DECT, which can be underestimated because of x-ray beam hardening, is still clinically reliable as regards the relative values for iodine identification. However, a more detailed examination is required before VNC images can be used as the equivalent of plain CT images.

## Data Availability

All data generated or analyzed during this study are included in this published article.

## Abbreviations

CT: Computed tomography
DECT: Dual-energy CT
ROI: Region of interest
VME: Virtual monoenergetic
VNC: Virtual non-contrast
